# Effect of different attachment configurations and skeletal anchorage force application points on canines during distalization of maxillary second molars with clear aligners: a finite element study

**DOI:** 10.1590/2177-6709.30.5.e252564.oar

**Published:** 2026-01-09

**Authors:** Nathalia de Oliveira DOMINGOS, Douglas Teixeira da SILVA, Ki Beom KIM, Weber José da Silva URSI, Guilherme de Araújo ALMEIDA

**Affiliations:** 1 Federal University of Uberlândia, Dental School, Department of Pediatric Dentistry (Uberlândia/MG, Brazil).; 2 Saint Louis University, Center of Advanced Dentistry Education, Orthodontic Department (Saint Louis/MI, USA).; 3 São Paulo State University, Dental School, Department of Operative Dentistry and Dental Materials (São José dos Campos/SP, Brazil).

**Keywords:** Orthodontic appliances, removable, Malocclusion, Angle Class II, Finite element analysis, Aparelhos ortodônticos removíveis, Má oclusão Classe II de Angle, Análise de elementos finitos

## Abstract

**Objective:**

This study investigated the influence of attachments on the maxillary posterior teeth using aligners and extra alveolar skeletal anchorage applied to precision cuts (PC) or buttons (BT) on the upper canines.

**Methods:**

3D virtual models were created from a tomography of a young adult Class II, division 1 Caucasian patient with healthy and non-restored full complement of permanent teeth (except third molars) was used. Six finite element models of the maxillary teeth with aligners and extra alveolar screw anchorage to simulate second molar distalization with three attachment configurations were used (NA, no attachments; VA, vertical attachments; VHA, vertical and horizontal attachments). PC or BT was used to transfer a anchoring force of 1.66 Newtons (N) on the canines. A 0.2mm of distalization activation was used between molars. Tooth displacements were measured in millimeters (mm) at the cusp tip of the second molar, canine, and central incisor in the coronal (X), sagittal (Y), and vertical (Z) axes.

**Results:**

The upper second molar distalized in all models resulting in anchorage loss of anterior teeth. In models without and with attachments, the distalization/anchorage loss ratio was 5:1 and 4:1, respectively. In most models, the teeth evaluated intruded and moved towards the midsagittal plane. The exception was model 3 (PC + VA), in which the molars were extruded.

**Conclusions:**

The distalization of upper second molars results in anchorage loss, whether attachments are present or not, despite the presence of skeletal anchorage. Its occurrence tends to be lower in the absence of attachments.

## RESUMO

**Objetivo:**

Este estudo investigou a influência de *attachments* nos dentes posteriores superiores usando alinhadores e ancoragem esquelética extra alveolar, combinada com *precision cuts* (PC) ou botões (BT) nos caninos superiores.

**Métodos:**

Modelos virtuais 3D foram criados a partir de uma tomografia de um paciente adulto jovem, caucasiano, com dentição permanente intacta desde os segundos molares e apresentando má oclusão de Classe II, divisão 1. Foram utilizados seis modelos de elementos finitos da arcada superior, contendo alinhadores e parafusos extra-alveolares entre os molares superiores, para simular a distalização dos segundos molares com três configurações de fixação (SA, sem *attachments*; AV, *attachments* verticais; AVH, *attachments* verticais e horizontais). PC ou BT foi utilizado para inserir uma força de ancoragem de 1,66 Newtons (N) nos caninos. Foi empregada uma ativação de distalização de 0,2mm entre os molares. Os deslocamentos dentários foram medidos em milímetros (mm) na ponta da cúspide do segundo molar, canino e incisivo central nos eixos sagital (Y), vertical (Z) e coronal (X).

**Resultados:**

O segundo molar superior distalizou em todos os modelos e os dentes anteriores perderam ancoragem. Nos modelos sem e com *attachments*, a relação distalização/perda de ancoragem foi de quase 5:1 e 4:1, respectivamente. Na maioria dos modelos, os dentes avaliados intruíram e moveram-se em direção ao plano sagital. A exceção foi o modelo 3 (PC + AV), no qual os molares foram extruídos.

**Conclusões:**

A perda de ancoragem ocorre durante a distalização do segundo molar superior, com ou sem *attachments*, mesmo na presença de ancoragem esquelética. Sua ocorrência tende a ser menor na ausência de *attachments*.

## INTRODUCTION

Currently, aligners are widely used to treat a variety of malocclusions. Among these is the Class II dentoalveolar malocclusion, which accounts for approximately half of all cases in Brazilians.^1^ There have been two treatment options available in these cases: extraction of the upper premolars (first or second) or distalization of the upper posterior teeth. When this last option is chosen, ideally posterior teeth should displace in the distal direction with minimal tipping and anchorage loss of the anterior teeth.^2^

Several studies^3^ ^,^ ^4^ have demonstrated the correction of Class II malocclusion using aligners, associated with previous hybrid distalization mechanics or simultaneously combined with skeletal anchorage with a force applied to precision cuts or to buttons bonded on the upper canines. It has also been found that attachments can assist in aligner fit improving tooth movement control.^2^ Attachments improve the distribution of stresses on the teeth^5^ ^-^ ^9^ minimizing tipping and anterior anchorage loss. 

For upper molar distalization many attachment designs have been proposed by clinicians and aligner companies,^2^ ^,^ ^4^ ^,^ ^10^ ^-^ ^14^ but there is no consensus, even if they should be used at all. Some studies^5^ ^,^ ^6^ ^,^ ^12^ ^-^ ^16^ have evaluated the biomechanics of tooth movement with aligners and the influence of attachments on dental displacement patterns. However, none of them have evaluated what this paper tries to clarify: the effect of no attachments versus different attachment designs during upper second molar distalization involving the use of a skeletal anchorage force parallel to the occlusal plane,^17^ from extra-alveolar screws to precision cuts or to bonded buttons in canines. The methodology involved the finite element method (FEM) which evaluates the biomechanical effect of a given structure subjected to a force through its stress, displacement, or deformation.^16^ ^,^ ^18^ ^-^ ^19^

Using this technology, the aim of this study was to evaluate the influence of the absence or presence of different attachments designs on the upper second molars distalization associated an anchorage force from the extra-alveolar screws to the precision cuts or bonded buttons on canines.

## MATERIAL AND METHODS

This study was submitted and approved by the Research Ethics Committee of the Federal University of Uberlândia (UFU) (CAAE 68334822.0.0000.5152). A cone-beam computed tomography (CBCT) scan of an 18-year-old female patient with Class II, division 1 malocclusion and complete permanent dentition up to the second molars was selected from the image file of UFU. 

To analyze biomechanical behavior, the range of orthodontic tooth movement was measured in virtual numerical models. Three-dimensional (3D) finite element models of the maxillary arch were generated from the CBCT scan. DICOM files were exported to Invesalius CTI software (Renato Archer, Campinas, Sao Paulo, Brazil) for segmentation and reconstruction of the structures. The maxilla was segmented up to the height of the zygomatic bone. Different structures, including cortical bone, cancellous bone, enamel, and dentin, were delimited by image density.^18^ ^,^ ^19^ A 0.2-mm thick periodontal ligament was defined around the tooth roots using Boolean operations.^18^

After segmentation, the 3D triangular mesh surface of each maxillary structure was exported in STL format (Stereolithography). The aligner, precision cuts, hooks with 4mm of height, buttons, and rectangular attachments with 3 mm high, 1 mm thick, and 2 mm wide, were designed using 3-Matic software (version 18.0; Materialise, Leuven, Belgium). The STL file of the 12 x 2 mm mini-screw was provided by the manufacturer (extra-alveolar mini-screw #5593, Peclab, Belo Horizonte, Brazil). The extra-alveolar screws were positioned on the zygomatic crest, 11 mm above the mesiobuccal cusp of the maxillary second molar on both sides.^20^ The aligner thickness was set at 0.75 mm, exclusively covering the crowns of the maxillary teeth. Precision cuts and buttons were positioned in the center of the cervical-buccal region of the maxillary canines. 

The STL files were imported into Femap software (Siemens PLM Software, Plano, Texas, USA), and the volumetric meshing process of the model was performed with 10-node tetrahedral elements, resulting in an average of 775945 nodes and 490227 elements. The model was then imported into ANSYS software (ANSYS, Pennsylvania, USA) for structural analysis, maximum and minimum stress data, and displacement of dental structures. In defining the boundary conditions, a smooth contact interface was defined at the interfaces and the top of the bone structure, except for the palatine bone, which was rigidly fixed in the x, y, and z axes. The entire upper portion of the maxilla, apart from the palate.

The interfaces between the different structures were considered bonded to prevent relative movement along all interfaces of the model. On the other hand, the attachments, buttons, and hooks of each contact were considered rigid. Furthermore, the contact between the tooth crowns and the aligner is frictional, with a coefficient of friction of 0.2.^11^ All materials were considered linear-elastic, isotropic, and homogeneous. For each material, the Poisson’s ratio and modulus of elasticity obtained from the literature were used, as shown in Table 1. 

**Table 1:** Modulus of elasticity e Poisson’s ratio of materials.

Material ​	Modulus of elasticity	Poisson’s ratio	References
	(Mpa) ​	(v) ​	
Tooth ​	19 600 ​	0.30 ​	Gomez et al.^12^
Periodontal ligament ​	50 ​	0.45 ​	Patiño et al.^18^​
Cortical bone ​	13 700 ​	0.30 ​	Patiño et al.^18^​
Cancelous bone	1 370 ​	0.30 ​	Patiño et al.^18^​
Aligner	528 ​	0.36 ​	Gomez et al.^12^​
Attachments ​	12 500 ​	0.36 ​	Gomez et al.^12^​
Mini-screw	110 000 ​	0.30 ​	Patiño et al.^18^​
Button	206 000 ​	0.30 ​	Ji et al.^14^​

**Figure 1:**
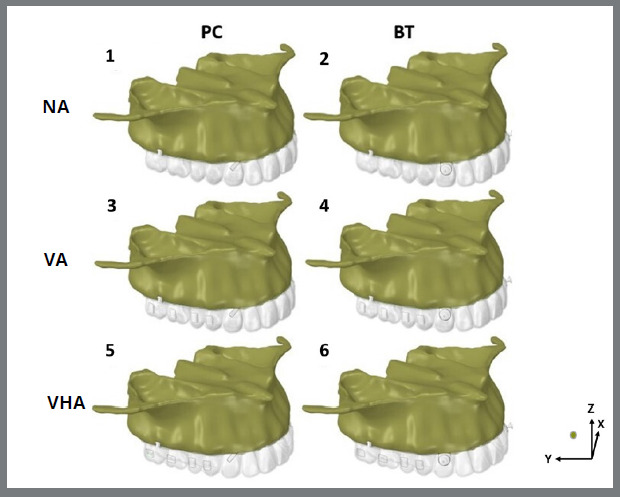
Configurations of finite element models used: NA - without attachments (models 1 and 2), VA = vertical attachments in premolars and molars (models 3 and 4), VHA = vertical attachments in premolars and horizontal in molars (models 5 and 6) and skeletal anchorage of extra-alveolar screws to precision cuts (models 1, 3 and 5) or buttons (models 2, 4 and 6). In addition to the models, the orientation X, Y, and Z axis used.

Six finite element models were generated (Fig. 1) according to the two factors under study: attachments at 3 levels (no attachments (NA), vertical attachments on molars and premolars (VA), and vertical attachments on the upper premolars and horizontal on the molars (VHA) and anchorage force from skeletal anchorage to the canines on precision cuts (PC) or bonded buttons (BT) (Fig. 1):

» Model 1: No attachments and anchorage from the extra-alveolar screws to precision cuts (NA + PC).
» Model 2: No attachments and anchorage from the extra-alveolar screws to buttons (NA + BT).
» Model 3: Vertical attachments on the upper premolars and molars and anchorage from the extra-alveolar screws to precision cuts (VA + PC).
» Model 4: Vertical attachments on the upper premolars and molars and anchorage from the extra-alveolar screws to buttons (VA + BT).
» Model 5: Vertical attachments on the upper premolars and horizontal on the molars and anchorage from the extra-alveolar screws to precision cuts (VHA + PC).
» Model 6: Vertical attachments on the upper premolars and horizontal on the molars and anchorage from the extra-alveolar screws to buttons (VHA + BT).

The models were subject to an anchorage force of 1.66 Newtons (N) from the extra-alveolar screws to the canines (precision cuts or bonded buttons), in addition to an initial distal displacement of 0.2 mm between upper first and second molars (Y axis).

Data analysis was performed comparatively according to the quantitative scales generated in the models in specific areas. The results were expressed using the Von Mises criterion, presenting maximum and minimum stresses to represent tensile and compressive forces, measured in Megapascal (MPa). Displacements were measured having as a reference the mesiobuccal cusp tip of the upper second molars, the cusp tip of the upper canines, and the incisal edge of the maxillary central incisors, and were expressed by displacement vectors, indicating the direction and magnitude of movement in the X (coronal), Y (sagittal), and Z (vertical) planes (Fig. 1).

## RESULTS

**Figure 2:**
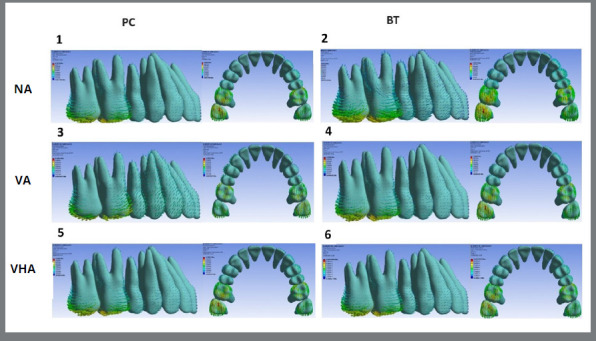
Lateral and occlusal views of the initial upper tooth movements according to models 1 to 6, expressed in vector arrows and measured in MPa.

**Figure 3:**
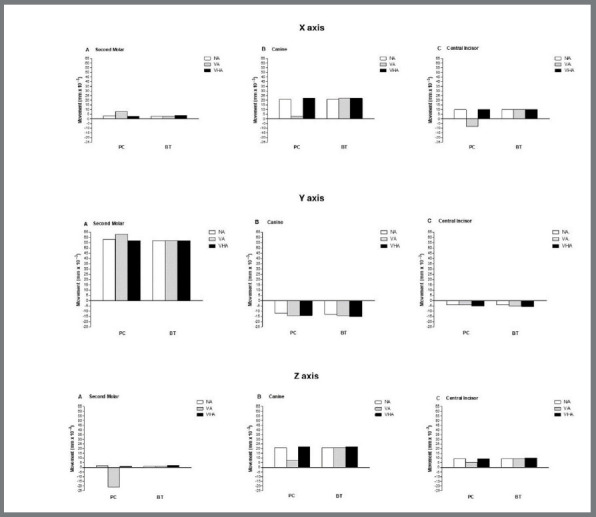
Comparison of coronal (X-axis), sagittal (Y-axis), and axial (Z-axis) tooth movement values of maxillary second molars, canines, and central incisors using precision cuts or buttons, and different attachment configurations (NA, VA, VHA), according to models 1 to 6. Positive values represent immediate displacement in the sagittal plane (X-axis), in the distal (Y-axis), and in the intrusion directions (Z-axis), and negative values represent the tendency of initial displacement of each axis in its respective opposite direction.

The initial displacement of the mesiobuccal cusp of the maxillary second molar, the cusp of the canine, and the incisal edge of the maxillary central incisor are shown in the lateral and occlusal (Fig. 2) views. Also, quantitative displacement and graphic depiction of the initial movement of the upper second molars, canines, and central incisors in the X, Y-axis, and Z are demonstrated in Table 2 and Figure 3. Finally, the ratio (%) between upper second molar distalization and canine and central incisor anchorage loss on the Y axis, according to each model, are shown in Table 3.

**Table 2:** Values (mm) of the initial displacement of the upper second molars, canines, and central incisors for models 1 to 6, in the X, Y, and Z-axes.

Model	Second molar			Canine			Central incisor		
	X	Y	Z	X	Y	Z	X	Y	Z
1 (PC + NA)	0.003	0.058	0.002	0.021	-0.012	0.021	0.01	-0.004	0.009
2 (BT + NA)	0.003	0.057	0.001	0.021	-0.013	0.021	0.01	-0.004	0.009
3 PC + VA)	0.008	0.063	-0.021	0.003	-0.014	0.007	-0.008	-0.004	0.005
4 (BT + VA)	0.003	0.057	0.001	0.022	-0.014	0.021	0.01	-0.005	0.009
5 (PC + VHA)	0.003	0.057	0.001	0.022	-0.014	0.022	0.01	-0.005	0.009
6 (BT + VHA)	0.004	0.057	0.002	0.022	-0.015	0.022	0.01	-0.006	0.01

On the X-axis, upper canines, central incisors, and second molars showed a similar displacement towards the midsagittal plane in almost all models. The exception was the model presenting exclusively vertical attachments and the anchorage force inserted on the precision cuts (model 3). In this model (PC + VA), the second molars displacement was slightly more significant than in the other models. Displacement towards the midsagittal plane suffered a significant reduction in the canines, while the upper incisors movement was in an opposite direction of the sagittal plane (Fig. 3, Table 2). 

On the Y axis, all models consistently presented second molar distalization in varying amounts and definite anchorage loss for canines and central incisors (Figs. 2 and 3, Table 2). Of the amount of total displacement, second molars were the ones that displaced most, followed by canines and central incisors (Figs. 2 and 3, Table 2). Without attachments, displacement of the second molars was similar in all models, except for model 3 (VA + PC), with smaller anchorage loss, regardless of where the anchorage force was applied (PC or BT). Specifically, in model 3, distal displacement was slightly more significant than in the other models (Fig. 3 and Table 2). When attachments were present, the results revealed the influence of their different configurations (VA and VHA) and the types of anchorage force insertions used (PC or BT), mainly on the loss of canine’s anchorage (models 3, 4, 5, and 6) and central incisors (models 4, 5 and 6) (Fig. 3, Table 2).

Table 3 shows the displacement ratio (%) of the upper second molars, canines, and central incisors compared to the total observed. Distal movement of the second molars was predominant, ranging from 73.45 to 78.38%. The loss of anchorage of the canines and central incisors ranged from 16.22 to 19.33% and from 4.94 to 7.22%, respectively. The greatest distal displacement of the second molars, accompanied by less anchorage loss of the central incisors and especially the canines, were obtained with the NA models, followed by the VA and VHA, respectively. Likewise, under this same reasoning, the best relationships between the predominance of distalization of the second molars and a lower loss of anchorage were obtained with NA independently if the anchorage force was inserted using PC or BT (Fig. 3, Table 3). 

**Table 3:** Percentual (%) comparison between the initial upper second molar movement, and canine and central incisor anchorage loss on the Y axis, according to each model.

​ Model	Upper teeth movement (%) ​			Anchorage loss (%)
	Second molar	Canine​	Central Incisor	Canine + central incisor
1 (PC + NA)​	+78.38%	-16.22%	-5.4%	-21.62%
2 (BT + NA)​	+78.38%	-16.22%	-5.4%	-21.62%
3 (PC + VA)​	+77.78%	-17.28%	-4.94%	-22.22%
4 (BT + VA)​	+75%​	-18.42%	-6.58%	-25%
5 (PC + VHA)​	+75%	-18.42%	-6.58%	-25%
6 (BT + VHA)​	+73.45%	-19.33%	-7.22%	-26.55%

In the Z axis, the movement was predominantly intrusive in the canines, central incisors, and second molars, in decreasing order (Fig. 1, 2, and 3; Table 2). The displacement tendency of these teeth was relatively similar, however slightly larger in the VHA models. The exception was model 3, which showed an evident reduction in the intrusive displacement of the canines and central incisors and an extrusion of the upper second molars (Fig. 3; Table 2).

## DISCUSSION

In this study, the aligner material was modeled with a Von Mises stress value of 528 MPa and a Poisson’s ratio of 0.36. These properties align closely with those of common thermoplastic materials, like polyurethane-based and PET-G-based aligners, which show similar elasticity and stress distribution.^14^ ^,^ ^17^ ^,^ ^18^ This comparison validates that the material assumptions in the finite element model accurately reflect clinically relevant aligner properties, enhancing the applicability of the findings.

Similar to some studies,^21^ ^,^ ^22^ ^,^ ^23^ the results indicated that the absence or presence of different attachment configurations in upper posterior teeth associated with different anchorage force points of application (PC or BT) in the upper canines, seems to influence the ratio between distalization of the upper second molar and anchorage the loss in the canines and central incisors (Figs. 2, and 3; Tables 2 and 3), even with skeletal anchorage.

However, our results do not corroborate other previous studies, where some did not find differences between the use or not of attachments^4^ ^,^ ^9^ ^,^ ^10^ ^,^ ^24^ and others that admitted an improvement in distalization as a whole when attachments were present.^2^ ^,^ ^3^ ^,^ ^6^ ^-^ ^8^ ^,^ ^18^ ^,^ ^23^ ^,^ ^25^ Probably, this divergence can be partially explained by the variability in the size, position, and shape of the attachments, the teeth on which they were positioned, the amount of activation used, the type of anchorage, and different sites of anchorage force application.^2^ ^,^ ^3^ ^,^ ^4^ ^,^ ^6^ ^-^ ^10^ ^,^ ^17^ ^,^ ^23^ ^-^ ^26^

According to our results, attachments reduced the immediate distal displacement of the second molar, and at the same time increased anchorage loss of the maxillary canines and incisors, especially when horizontal attachments were present on both molars. The best distalization ratio was obtained the models without attachments, regardless of whether the anchorage force was inserted in the PC or the BT. In both situations, this ratio was almost 5:1 (78.38%-21.32%) without attachments. On the other hand, when attachments were present in molars and premolars (Tables 2 and 3), this rate was reduced to 4:1 (75.31%-24.69%), mainly when the insertion of the anchorage force used was BT. It seems that the attachments acted as resistant force to the upper second molar displacement, taxing anchorage. Due to this effect, a question arises: should attachments be used in all clinical situations, to enhance control of individual tooth movement or to increase retention, or should them be used only in mechanical situations where the side effects of a given tooth movement are irrelevant or controllable? It has been shown^10^ ^,^ ^13^ ^,^ ^17^ ^,^ ^21^ ^,^ ^23^ that the loss of anchorage is an almost inevitable side effect of the distalization of upper molars. Some studies^21^ ^,^ ^23^ even state that as more teeth are included in the distalization mechanics (sequential distalization), more anchorage is lost, and the previously distalized second molars relapse. This data is not consensual in the literature, where some studies^3^ ^,^ ^22^ ^,^ ^26^ have indicated that sequential distalization presents less anchorage loss, around 12%, for displacements less than 1.5 mm. It is essential to highlight that anchorage loss of around 20-25% was verified after applying distalizing force restricted only to the upper second molars. Based on this, for non-extraction treatment of Class II cases in the permanent dentition it seems reasonable to restrict the distalization of upper posterior teeth to a limit of 1/3 of a Class II correction, seemingly prioritizing the individual movement of each posterior tooth or even considering some form of distalization mechanism before aligners. 

Again, this nearly consensual anchorage loss^10^ ^,^ ^13^ ^,^ ^17^ ^,^ ^21^ ^,^ ^23^ raises another question: why does this happen even with skeletal anchorage? Could it be because its use, magnitude, and direction of the force applied are rarely considered during the staging of aligners? Consequently, its application might alter the mechanical force system present in each aligner, favoring its deformation and possible loss of tracking? Unfortunately, this research couldn’t provide an answer to this question.

In models where attachments were used, distalization/anchorage loss ratio favored slightly precision cuts (PC) compared to bonded buttons (BT), which is not in agreement other publications.^14^ ^,^ ^25^ Although force application in the precision cuts has been attributed to increasing aligner deformation;^6^ ^,^ ^17^ ^,^ ^25^ in this study, distalization restricted to evaluating second molars could not identify its possible influence.

In general, except in model 3 (VA + PC), concomitant with the displacement of the second molar distally and anchorage loss of canines and central incisors (Figs. 2 and 3, Table 2), there was a relative intrusion of both ^10^ ^-^ ^15^ ^,^ ^18^ (Fig. 3, Table 2) and a slight immediate displacement towards the midsagittal plane (Fig. 3, Table 2) regardless of the absence or configuration of attachments or the type of insertion of the anchorage force in the canines (PC or BT).

These results, similar to previous studies,^10^ ^-^ ^15^ ^,^ ^22^ ^,^ ^24^ suggest reasonable vertical control at the beginning of the distalization of second molars and the need of expansion to be incorporated in the virtual treatment plan. Model 3 presented distinct displacements compared to the other models (Fig. 3). The second molars showed a slight increase in their initial movement towards the distal and to the midsagittal plane, accompanied by undesirable extrusion. Canines presented less displacement towards the midsagittal plane and in intrusion. Central incisors also presented less intrusion; however, accompanied by displacement opposite to the midsagittal plane. One can speculate that the differences of model 3 (VA+PC) in relation to the others, especially to model 4, are a consequence of the deformation of the aligner^6^ ^,^ ^7^ ^,^ ^26^ in the region of the PC. One possibility is that the application of vertical attachments increases the resistance to second molars displacement, deforming PC in the buccal direction, reducing the displacement of the canines towards the sagittal plane, and moving the crown of the central incisor in the opposite direction to the midsagittal plane.

This study presents limitations as it is based on the initial movement of the evaluated teeth through the study of finite elements. As it is a laboratory study, it does not include variables such as bite force, the presence of saliva, patient cooperation, or the periodontal condition present. Therefore, new clinical studies evaluating different mechanical options for the distalization of posterior-superior teeth, preferably randomized, are necessary.

## CONCLUSION

Even in the presence of skeletal anchorage, anchorage loss occurs during distalization of the upper second molar using clear aligners, regardless of whether attachments are present. Additionally, it is less likely to be observed in setups without attachments, regardless of whether the anchoring force is applied to precision cuts or to bonded buttons.
